# Liver X receptors regulate hepatic F4/80^**+**^CD11b^+^ Kupffer cells/macrophages and innate immune responses in mice

**DOI:** 10.1038/s41598-018-27615-7

**Published:** 2018-06-18

**Authors:** Kaori Endo-Umeda, Hiroyuki Nakashima, Shihoko Komine-Aizawa, Naoki Umeda, Shuhji Seki, Makoto Makishima

**Affiliations:** 1 Division of Biochemistry, Department of Biomedical Sciences, Nihon University School of Medicine, 30-1 Oyaguchi-kamicho, Itabashi-ku, Tokyo, 173-8610 Japan; 2 Department of Immunology and Microbiology, National Defense Medical College, 3-2 Namiki, Tokorozawa, Saitama, 359-8513 Japan; 3 Division of Microbiology, Department of Pathology and Microbiology, Nihon University School of Medicine, 30-1 Oyaguchi-kamicho, Itabashi-ku, Tokyo, 173-8610 Japan

## Abstract

The liver X receptors (LXRs), LXRα and LXRβ, are nuclear receptors that regulate lipid homeostasis. LXRs also regulate inflammatory responses in cultured macrophages. However, the role of LXRs in hepatic immune cells remains poorly characterized. We investigated the role of LXRs in regulation of inflammatory responses of hepatic mononuclear cells (MNCs) in mice. Both LXRα and LXRβ were expressed in mouse hepatic MNCs and F4/80^+^ Kupffer cells/macrophages. LXRα/β-knockout (KO) mice had an increased number of hepatic MNCs and elevated expression of macrophage surface markers and inflammatory cytokines compared to wild-type (WT) mice. Among MNCs, F4/80^+^CD11b^+^ cells, not F4/80^+^CD11b^−^ or F4/80^+^CD68^+^ cells, were increased in LXRα/β-KO mice more than WT mice. Isolated hepatic MNCs and F4/80^+^CD11b^+^ cells of LXRα/β-KO mice showed enhanced production of inflammatory cytokines after stimulation by lipopolysaccharide or CpG-DNA compared to WT cells, and LXR ligand treatment suppressed lipopolysaccharide-induced cytokine expression in hepatic MNCs. Lipopolysaccharide administration also stimulated inflammatory cytokine production in LXRα/β-KO mice more effectively than WT mice. Thus, LXR deletion enhances recruitment of F4/80^+^CD11b^+^ Kupffer cells/macrophages and acute immune responses in the liver. LXRs regulate the Kupffer cell/macrophage population and innate immune and inflammatory responses in mouse liver.

## Introduction

Various immune cell types are abundantly present in the liver and play important roles in regulating immunity and inflammation^1,2^. The adult liver is composed of hepatocytes and other non-parenchymal cells including immune cells such as Kupffer cells/macrophages, natural killer cells, natural killer T cells, T lymphocytes, B lymphocytes, hepatic stellate cells, and liver sinusoidal endothelial cells. Kupffer cells/macrophages are classified into two subsets, resident Kupffer cells and bone marrow-derived Kupffer cells/macrophages^3,4^. Resident Kupffer cells are yolk sac-derived, radio-resistant, express F4/80 plus CD68 on the cell surface, and show high phagocytic activity and reactive oxygen species production^1,5,6^. On the other hands, bone marrow-derived Kupffer cells/macrophages are radio-sensitive, express F4/80 plus CD11b, and are involved in acute inflammatory reactions and liver regeneration^3,7–9^. High cholesterol diet increases F4/80^+^CD11b^+^ Kupffer cells/macrophages and inflammatory cytokine production in the mouse liver^10^. Inflammatory cytokine production and acute liver injury after treatment with α-galactosylceramide or CpG-DNA are exacerbated in hypercholesterolemic mice^11^. These findings indicate that cholesterol metabolism is associated with hepatic innate immune responses.

The liver X receptors (LXRs), LXRα and LXRβ, are members of the nuclear receptor superfamily of ligand-dependent transcription factors. Whereas LXRα is preferentially expressed in the liver, adipose tissue, small intestine and macrophage, LXRβ is widely present throughout the whole body^12^. Both LXRs are activated by oxysterols, such as 24,25(*S*)-epoxycholesterol and 22(*R*)-hydroxycholesterol, and regulate expression of genes involved in lipid metabolism, such as ATP-binding cassette A1, cholesterol 7α-hydroxylase, and sterol regulatory element-binding protein 1c^12^. In addition, LXRs regulate immune responses in immune cells, such as macrophages and dendritic cells^13^. In murine peritoneal macrophages, LXR activation suppresses acute inflammatory cytokine expression mediated by Toll-like receptor (TLR) 4 by inhibiting nuclear factor-κB transactivation, a mechanism called transrepression^14,15^. In contrast, LXR agonist treatment induces TLR4 expression and enhances inflammatory responses induced by lipopolysaccharide (LPS) in human blood monocyte-derived macrophages^16^. Emerging evidence indicates that macrophages are developed and function in a tissue-specific manner^17–19^. Furthermore, hepatic stellate cells are activated in chronic liver injury and cause severe liver fibrosis in LXRα/β-knockout (KO) mice compared to wild-type (WT) mice^20^. Liver sinusoidal endothelial cells are also involved in liver fibrosis and LXRα deletion exacerbates liver inflammation and fibrosis in a mouse model^21^. Although the metabolic function of LXRs in hepatocytes has been well investigated, the role of LXRs in hepatic immune cells, specifically Kupffer cells/macrophages, remains poorly understood. In this study, we examined the role of hepatic mononuclear cells (MNCs) using LXRα/β-KO mice and found that LXRs regulate the composition of the immune cell population and innate immune responses in the liver.

## Results

### LXRs are expressed functionally in mouse hepatic MNCs

**Figure 1:**
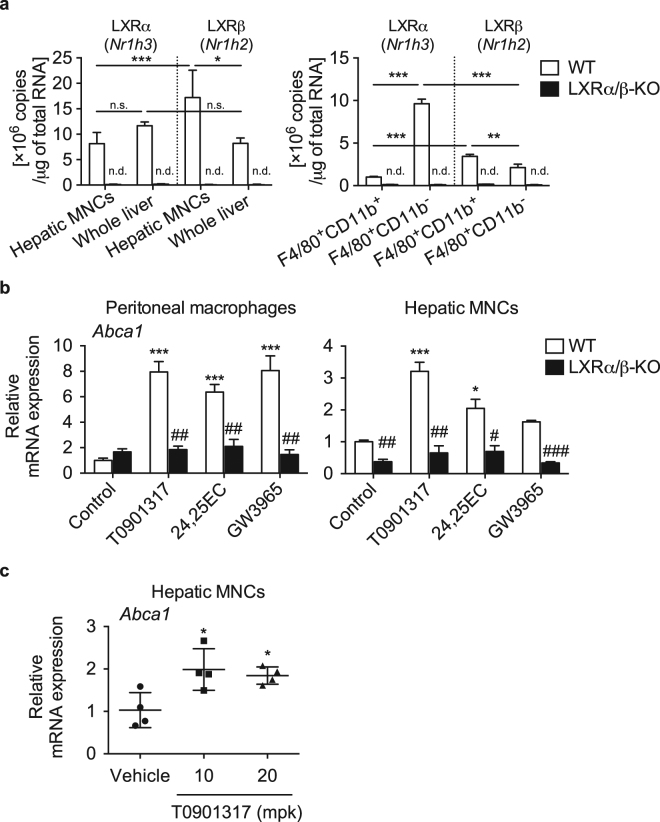
Expression and function of LXRs in hepatic MNCs. (**a**) Hepatic MNCs were isolated from the liver of WT mice, and mRNA copy numbers of LXRα (*Nr1h3)* and LXRβ (*Nr1h2*) were compared to those in whole liver samples (n = 8 for each group). LXRα (*Nr1h3)* and LXRβ (*Nr1h2*) mRNA levels were also evaluated in isolated F4/80^+^CD11b^+^ and F4/80^+^CD11b^−^ cells (n = 4 for each group). n.d., not detected. *P < 0.05; **P < 0.01; ***P < 0.001; n.s., not significant (one-way ANOVA followed by Tukey’s multiple comparisons for 4 groups of WT samples). (**b**) Thioglycolate elucidated-peritoneal macrophages and hepatic MNCs isolated were isolated from WT and LXRα/β-KO mice and were treated with vehicle control (ethanol), T0901317 (1 μM), 24,25(*S*)-epoxycholesterol (24,25EC) (10 μM), or GW3965 (1 μM) for 18 hours. *Abca1* mRNA levels were quantified (n = 3 for each group). *P < 0.05; ***P < 0.001 compared to Control (one-way ANOVA followed by Tukey’s multiple comparisons); ^#^*P* < 0.05; ^##^*P* < 0.01, ^###^*P* < 0.001 compared to WT (Student’s *t* test). (**c**) WT mice were fed a diet containing vehicle control (corn oil), or T0901317 (10 or 20 mg/kg/day (mpk)) for 7 days. *Abca1* mRNA levels in hepatic MNCs were examined (n = 4 for each group). *P < 0.05 compared to control (one-way ANOVA followed by Tukey’s multiple comparisons). mRNA values were normalized with *Ppib* (**a**) or *Gapdh* (**b**,**c**) mRNA levels.

To examine the expression and function of LXRα and LXRβ in immune cells in the liver, we isolated hepatic MNCs by collagenase digestion and Percoll gradient centrifugation. First, we evaluated mRNA levels of LXRα (gene symbol *Nr1h3*) and LXRβ (*Nr1h2*) in hepatic MNCs and in whole liver. We detected both LXRα and LXRβ mRNA expression in hepatic MNCs and whole liver from WT mice but not from LXRα/β-KO mice (Fig. 1a). While LXRα mRNA levels in hepatic MNCs were similar to those in the whole liver, LXRβ was at higher levels than LXRα in hepatic MNCs and more abundantly in hepatic MNCs than in whole liver. We also evaluated mRNA expression of LXRα and LXRβ in F4/80^+^ Kupffer cells/macrophages isolated by fluorescence-activated cell sorting (FACS) and detected expression of both LXRs in WT cells but not in LXRα/β-KO cells (Fig. 1a). LXRα was more highly expressed in F4/80^+^CD11b^−^ cells than in F4/80^+^CD11b^+^ cells, while LXRβ expression was slightly higher in F4/80^+^CD11b^+^ cells. While LXRα expression was higher than LXRβ in F4/80^+^CD11b^−^ cells, LXRβ levels were slightly higher than LXRα levels in F4/80^+^CD11b^+^ cells. Next, we examined the effect of LXR ligand on target gene expression in hepatic MNCs with comparison to that in peritoneal macrophages. We treated cells with a synthetic LXR ligand (T0901317 or GW3965), or a potent natural ligand (24,25(*S*)-epoxycholesterol). All LXR ligands effectively increased mRNA levels of *Abca1*, which encodes the ABC-binding cassette transporter A1, in peritoneal macrophages isolated from WT mice, and in hepatic MNCs from WT mice although the effect of GW3965 was not statistically significant (Fig. 1b). The effects of LXR ligands were abolished in peritoneal macrophages and hepatic MNCs from LXRα/β-KO mice (Fig. 1b). LXR ligand effect was further examined *in vivo* in WT mice fed a diet containing T0901317 for 1 week. Expression of *Abca1* was significantly induced in hepatic MNCs isolated from mice administered T0901317 (Fig. 1c). These results indicate that LXRs are expressed functionally in hepatic MNCs.

### Hepatic MNCs and F4/80^+^CD11b^+^ macrophages are increased in LXRα/β-KO liver

**Figure 2:**
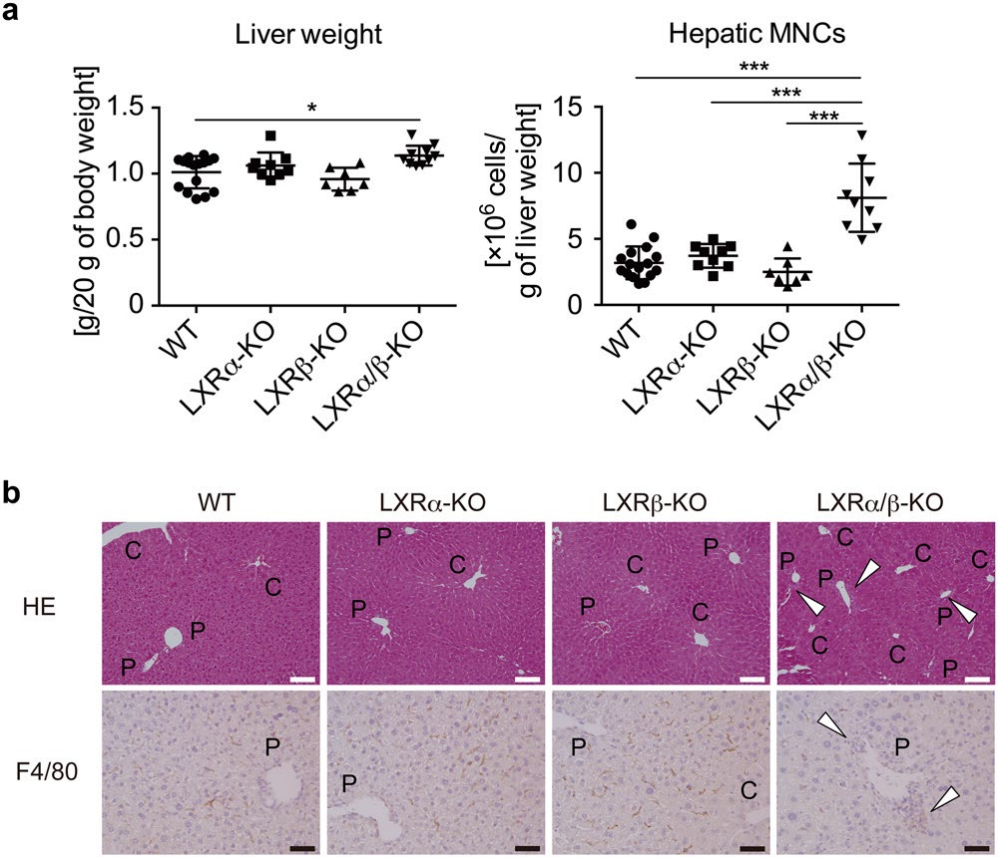
Increased hepatic MNCs in LXR-deficient mice. (**a**) Liver weight and hepatic MNC number in WT, LXRα-KO, LXRβ-KO, and LXRα/β-KO mice. WT, n = 16; LXRα-KO, n = 9; LXRβ-KO, n = 7; LXRα/β-KO, n = 10. *P < 0.05; ***P < 0.001 (one-way ANOVA followed by Tukey’s multiple comparisons). (**b**) Hematoxylin and eosin staining and F4/80 immunostaining of the liver samples from WT, LXRα-KO, LXRβ-KO, and LXRα/β-KO mice. The open triangles indicate accumulated MNCs. C and P indicate central vein and portal vein, respectively. C, central vein; P, portal vein. Scale bar = 100 μm (white) or 50 μm (black).

We examined the role of LXRs in composition of hepatic immune cell populations by comparing hepatic MNCs from WT, LXRα-KO, LXRβ-KO, and LXRα/β-KO mice. Liver weight of LXRα/β-KO mice was slightly increased compared to WT mice (Fig. 2a). Interestingly, total hepatic MNC number in LXRα/β-KO mice was about 2.5-fold compared to those in other groups (Fig. 2a). Liver histology showed that more MNCs were accumulated in periportal areas of LXRα/β-KO mice and that F4/80^+^ cells were also increased in these areas of LXRα/β-KO mice (Fig. 2b).

**Figure 3:**
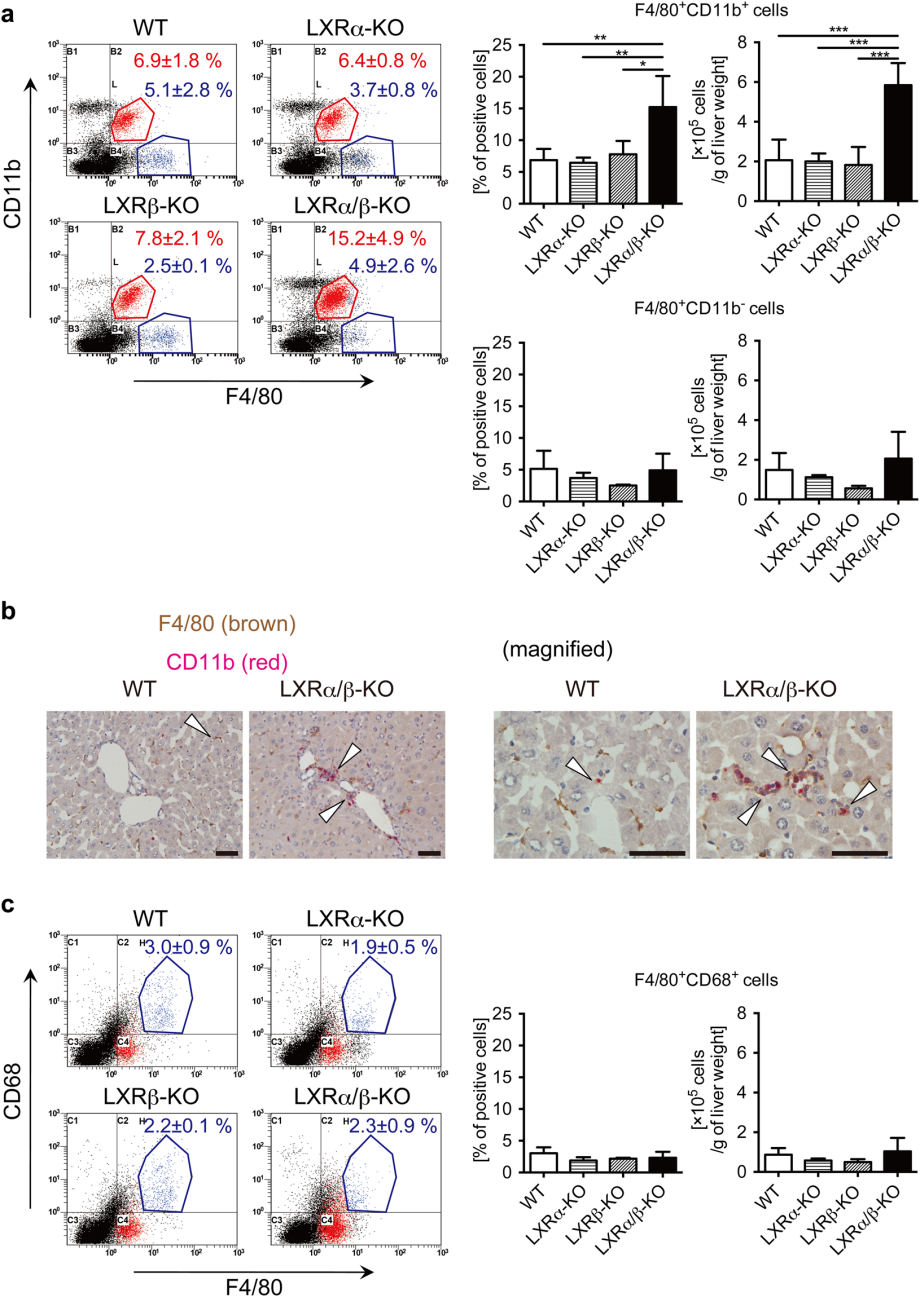
Increased population of F4/80^+^CD11b^+^ Kupffer cells/macrophages in the liver of LXRα/β-KO mice. (**a**) Representative flow cytometry for F4/80 and CD11b staining. Percentages and numbers of F4/80^+^CD11b^+^ cells and F4/80^+^CD11b^−^ cells were shown in right graphs. (**b**) Double immunostaining of F4/80 (brown) and CD11b (red) in the liver of WT and LXRα/β-KO mice. The open triangles indicate F4/80^+^CD11b^+^ cells. Scale bar = 50 μm. (**c**) Representative flow cytometry for F4/80 and CD68 staining. Percentages and numbers of F4/80^+^CD68^+^ cells were shown in right graphs. Hepatic MNCs were isolated from WT, LXRα-KO, LXRβ-KO, and LXRα/β-KO mice, stained with FITC-conjugated anti-F4/80, PE-Cy5-conjugated anti-CD11b, biotin-conjugated anti-CD68 and PE-streptavidin, and examined with flow cytometric analysis (**a**,**c**: WT, n = 6; LXRα-KO, n = 5; LXRβ-KO, n = 3; LXRα/β-KO, n = 4). *P < 0.05; **P < 0.01; ***P < 0.001 (one-way ANOVA followed by Tukey’s multiple comparisons).

To identify the immune cell types increased in the liver of LXRα/β-KO mice, we performed flow cytometric analysis. We found that the percentage and number of F4/80^+^CD11b^+^ cells but not of F4/80^+^CD11b^−^ cells were increased in LXRα/β-KO mice compared to WT mice (Fig. 3a). Double immunostaining also showed that F4/80^+^CD11b^+^ cells were increased in the liver of LXRα/β-KO mice (Fig. 3b and Supplementary Fig. 1). The percentage and number of F4/80^+^CD68^+^ cells were not increased in LXRα/β-KO mice (Fig. 3c). These results indicate that F4/80^+^CD11b^+^ Kupffer cells/macrophages are increased in the liver of LXRα/β-KO mice.

### Altered lipid content in plasma and liver of LXRα/β-KO mice

**Figure 4:**
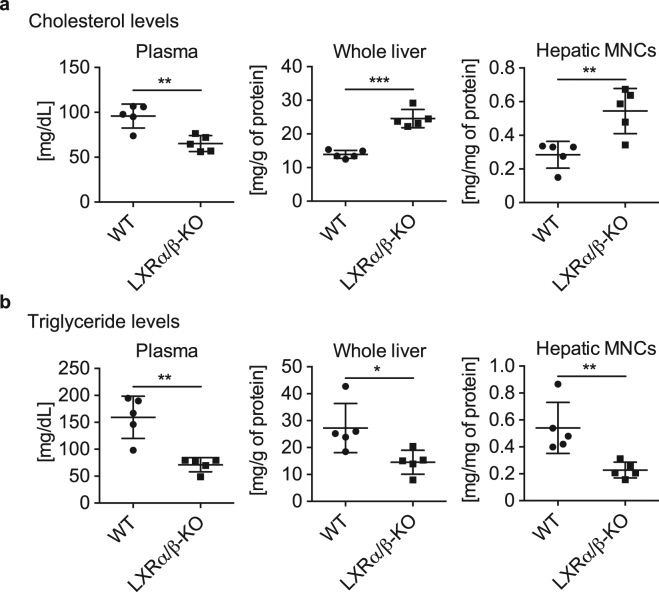
Dysregulated lipid metabolism in whole liver and hepatic MNCs of LXRα/β-KO mice. Measurements of cholesterol levels (**a**) and triglyceride levels (**b**) in plasma, whole liver, and hepatic MNCs from WT and LXRα/β-KO mice (n = 5 for each group). *P < 0.05; **P < 0.01; ***P < 0.001 (Student’s *t* test).

We previously reported that high cholesterol diet increases F4/80^+^CD11b^+^ Kupffer cells/macrophages in the liver of mice^10^. We examined whether increased hepatic cholesterol levels are associated with recruitment of F4/80^+^CD11b^+^ Kupffer cells/macrophages in the liver of LXRα/β-KO mice. Plasma cholesterol levels were lower and hepatic cholesterol levels were higher in LXRα/β-KO mice that WT mice (Fig. 4a), consistent with previous reports^22,23^. Cholesterol levels were also elevated in hepatic MNCs of LXRα/β-KO mice (Fig. 4a). On the other hand, triglyceride levels were reduced in plasma, whole liver and hepatic MNCs of LXRα/β-KO mice compared to WT mice (Fig. 4b). Thus, lipid metabolism is dysregulated in immune cells in the liver of LXRα/β-KO mice.

### LXR deletion increases M1-macrophage markers in isolated hepatic MNCs and *Ccl2* mRNA expression in the liver parenchyma

**Figure 5:**
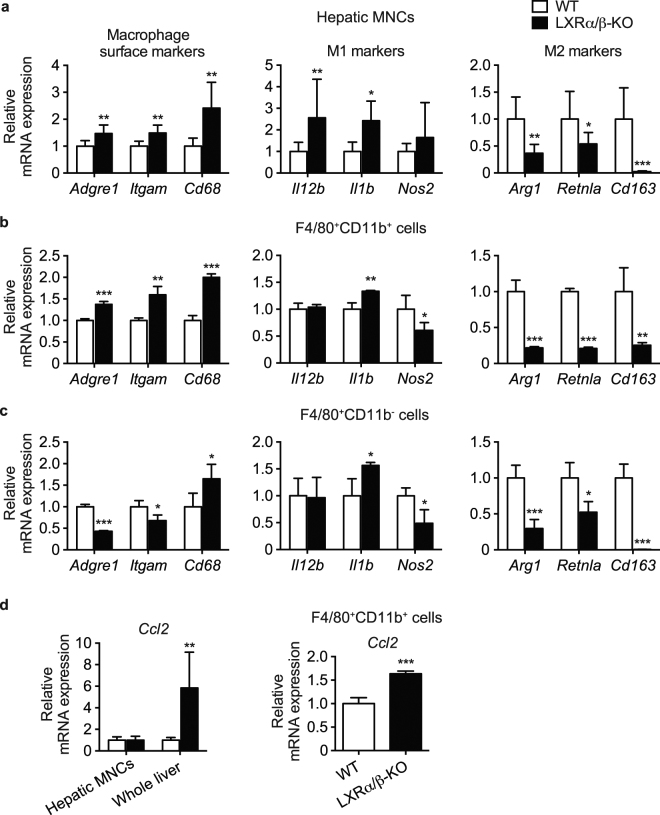
Elevated expression of M1 macrophage markers in hepatic MNCs and F4/80^+^ Kupffer cells/macrophages of LXRα/β-KO mice. mRNA expression of macrophage surface markers (*Adgre1*, *Itgam*, *Cd68*), M1 and M2 macrophage markers (M1: *Il12b*, *Il1b*, *Nos2*; M2: *Arg1*, *Retnla*, *Cd163*) in hepatic MNCs (**a**), F4/80^+^CD11b^+^ cells (**b**), and F4/80^+^CD11b^−^ cells (**c**) were evaluated (MNCs, n = 8; F4/80^+^CD11b^+^ cells, n = 4; F4/80^+^CD11b^−^ cells, n = 4 for each genotype). mRNA expression of monocyte chemoattractant protein-1 (*Ccl2*) (**d**) in hepatic MNCs, whole liver and F4/80^+^CD11b^+^ cells from WT and LXRα/β-KO mice were also examined (MNCs, n = 8; whole liver, n = 8; F4/80^+^CD11b^+^ cells, n = 4 for each genotype). mRNA levels were quantified and normalized with *Gapdh* or *Ppib* mRNA levels. *P < 0.05; **P < 0.01; ***P < 0.001 compared to WT (Student’s *t* test).

We performed gene expression analysis in hepatic MNCs isolated from WT and LXRα/β-KO mice. Expression of common macrophage marker genes, F4/80 (gene symbol, *Adgre1*), CD11b (*Itgam*) and CD68 (*Cd68*), was significantly elevated in hepatic MNCs of LXRα/β-KO mice (Fig. 5a). Macrophages are divided into two types, proinflammatory M1 macrophages and anti-inflammatory M2 macrophages^24^. M1 marker genes, interleukin-12B (IL-12B, *Il12b*), IL-1B (*Il1b*) and nitric oxide synthase 2 (*Nos2*), were increased whereas M2 marker genes, arginase 1 (*Arg1*), resistin-like molecule α (*Retnla*) and CD163 (*Cd163*), were reduced in hepatic MNCs of LXRα/β-KO mice (Fig. 5a). Next, we isolated F4/80^+^CD11b^+^ and F4/80^+^CD11b^−^ Kupffer cells/macrophages from WT and LXRα/β-KO hepatic MNCs by FACS and performed gene expression analysis. Similar to hepatic MNCs, expression of common macrophage marker genes, *Adgre1*, *Itgam* and *Cd68*, was increased in LXRα/β-KO F4/80^+^CD11b^+^ cells compared to WT cells (Fig. 5b). While *Il1b* expression was increased and *Nos2* was decreased, all M2 marker genes, *Arg1*, *Retnla* and *Cd163*, were decreased in LXRα/β-KO F4/80^+^CD11b^+^ cells (Fig. 5b). Similar changes in M1 marker genes and M2 marker genes were also observed in LXRα/β-KO F4/80^+^CD11b^−^ cells, but these KO cells had decreased expression of *Adgre1* and *Itgam* and increased *Cd68* expression compared to WT F4/80^+^CD11b^−^ cells (Fig. 5c). We evaluated mRNA levels of chemokine (C-C motif) ligand 2 (*Ccl2*, also called monocyte chemoattractant protein-1) in hepatic MNCs and whole liver from WT and LXRα/β-KO mice. The *Ccl2* gene encodes monocyte chemoattractant protein-1, which recruits monocytes/macrophages from bone marrow to peripheral tissues including liver and is involved in the pathogenesis of liver injury^25,26^. While expression levels of *Ccl2* in hepatic MNCs were similar between WT and LXRα/β-KO mice, it was highly expressed in the whole liver of LXRα/β-KO mice compared to WT mice (Fig. 5d). Interestingly, *Ccl2* mRNA levels in LXRα/β-KO F4/80^+^CD11b^+^ Kupffer cells/macrophages were slightly higher than in WT cells (Fig. 5d). These results suggest that monocyte chemoattractant protein-1 activity is increased in hepatic cell components other than MNCs and also in F4/80^+^CD11b^+^ Kupffer cells/macrophages in the liver of LXRα/β-KO mice.

### Effect of LXR activation and LXR deletion on inflammatory responses in isolated hepatic MNCs

**Figure 6:**
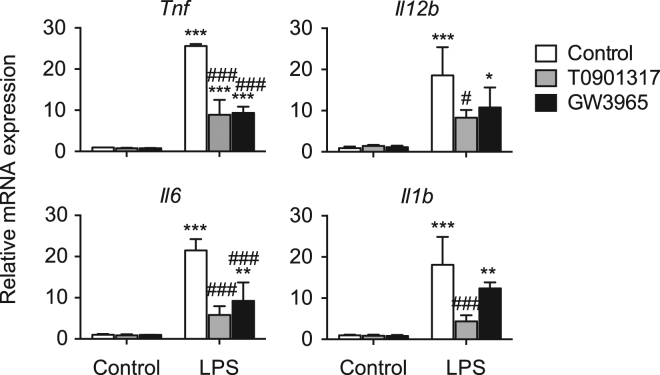
LXR ligand activation suppresses LPS-induced pro-inflammatory cytokine expression in hepatic MNCs. Hepatic MNCs were isolated from WT mice and treated with vehicle control, T0901317 (1 μM) or GW3965 (1 μM) for 18 hours, and then stimulated without or with LPS (1 ng/mL) for 3 hours. mRNA levels of *Tnf*, *Il12b*, *Il6* and *Il1b* were quantified and normalized with *Gapdh* mRNA levels (n = 3 for each group). *P < 0.05; **P < 0.01; ***P < 0.001 compared to LPS-unstimulated control; ^#^*P* < 0.05; ^###^*P* < 0.001 compared to LPS-stimulated control (one-way ANOVA followed by Tukey’s multiple comparisons).

To examine the effect of LXR ligand activation on acute inflammatory responses induced by LPS, a TLR4 ligand, in mouse hepatic MNCs, we isolated MNCs from WT mice and stimulated them with LPS after pretreatment with T0901317 or GW3965. LXR ligand treatments repressed LPS-induced expression of inflammatory cytokine genes, tumor necrosis factor α (TNF-α, *Tnf*), *Il12b*, *Il6*, and *Il1b* (Fig. 6). Thus, LXR activation suppresses the expression of inflammatory genes in hepatic MNCs.

**Figure 7:**
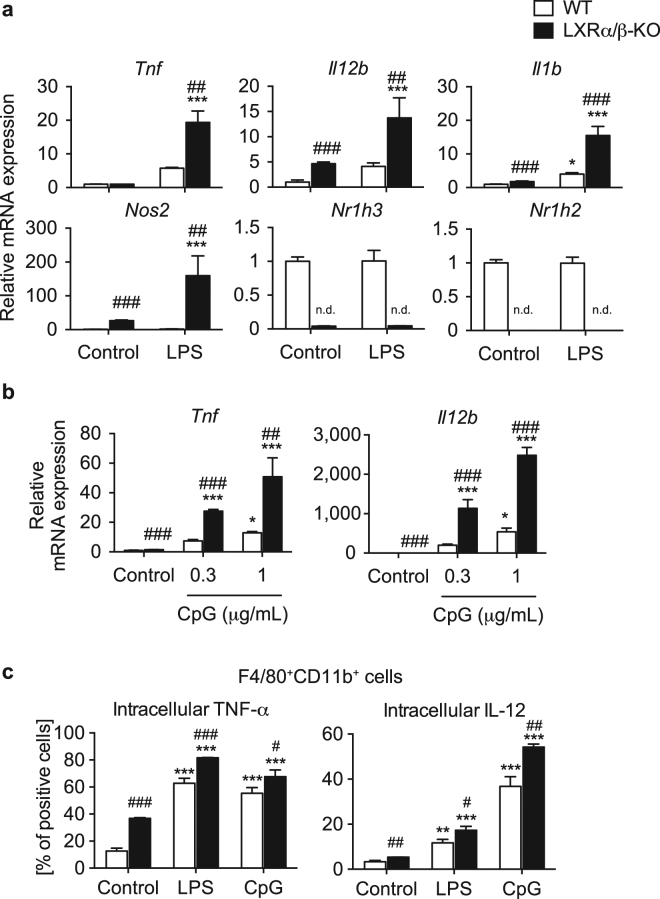
LXR-deficient hepatic MNCs enhance inflammatory gene expression and LXR deletion increases TNF-α and IL-12 levels in F4/80^+^CD11b^+^ cells. Hepatic MNCs were isolated from WT and LXRα/β-KO mice and stimulated without or with LPS (1 ng/mL) (**a**) or CpG-DNA (0.3 or 1 μg/mL) (**b**) for 3 hours. mRNA levels of *Tnf*, *Il12b*, *Il1b* and *Nos2* were quantified and normalized with *Gapdh* mRNA levels (n = 4 for each group). n.d., not detected. (**c**) Intracellular protein expression of TNF-α and IL-12 in F4/80^+^CD11b^+^ cells. Hepatic MNCs isolated from WT and LXRα/β-KO mice were stimulated without or with LPS (1 μg/mL) or CpG-DNA (10 μg/mL) for 4 hours, and the percentages of TNF-α positive cells and IL-12 positive cells in F4/80^+^CD11b^+^ cells were evaluated by flow cytometric analysis. *P < 0.05; **P < 0.01; ***P < 0.001 compared to control (one-way ANOVA followed by Tukey’s multiple comparisons); ^#^*P* < 0.05; ^##^*P* < 0.01; ^###^*P* < 0.001 compared to WT (Student’s *t* test).

LXR deletion has been reported to enhance inflammatory gene expression in peritoneal macrophages and intestinal epithelial cells^14,27^. Here, we examined the effect of LXR deletion on inflammatory gene-mediated acute inflammatory responses in hepatic MNCs. We isolated hepatic MNCs from WT and LXRα/β-KO mice and stimulated cells with LPS. Although LPS stimulation increased expression of *Tnf*, *Il12b* and *Il6* in WT MNCs (Fig. 7a), there are differences in the expression values compared to those shown in Fig. 6. These differences may be due to the complexity of experimental procedures for cell isolation. LPS stimulation increased mRNA levels of *Tnf*, *Il12b*, *Il1b* and *Nos2* in LXRα/β-KO MNCs more effectively than in WT MNCs (Fig. 7a). Treatment of CpG-DNA, a TLR9 ligand, increased mRNA expression of *Tnf* and *Il12b* in LXRα/β-KO MNCs more effectively than WT cells (Fig. 7b). We evaluated intracellular cytokine levels in F4/80^+^CD11b^+^ Kupffer cells/macrophages isolated from WT and LXRα/β-KO mice by FACS. In the absence of treatment with LPS or CpG, TNF-α positive cells and IL-12 positive cells were increased in LXRα/β-KO cells compared to WT cells (Fig. 7c). Stimulation with LPS or CpG increased TNF-α positive cells and IL-12 positive cells among WT F4/80^+^CD11b^+^ cells and was more effective in LXRα/β-KO cells (Fig. 7c). These results indicate that LXR deletion enhances inflammatory gene expression by TLR ligands.

### LXR deletion exacerbates LPS-induced acute hepatic inflammation in mice

**Figure 8:**
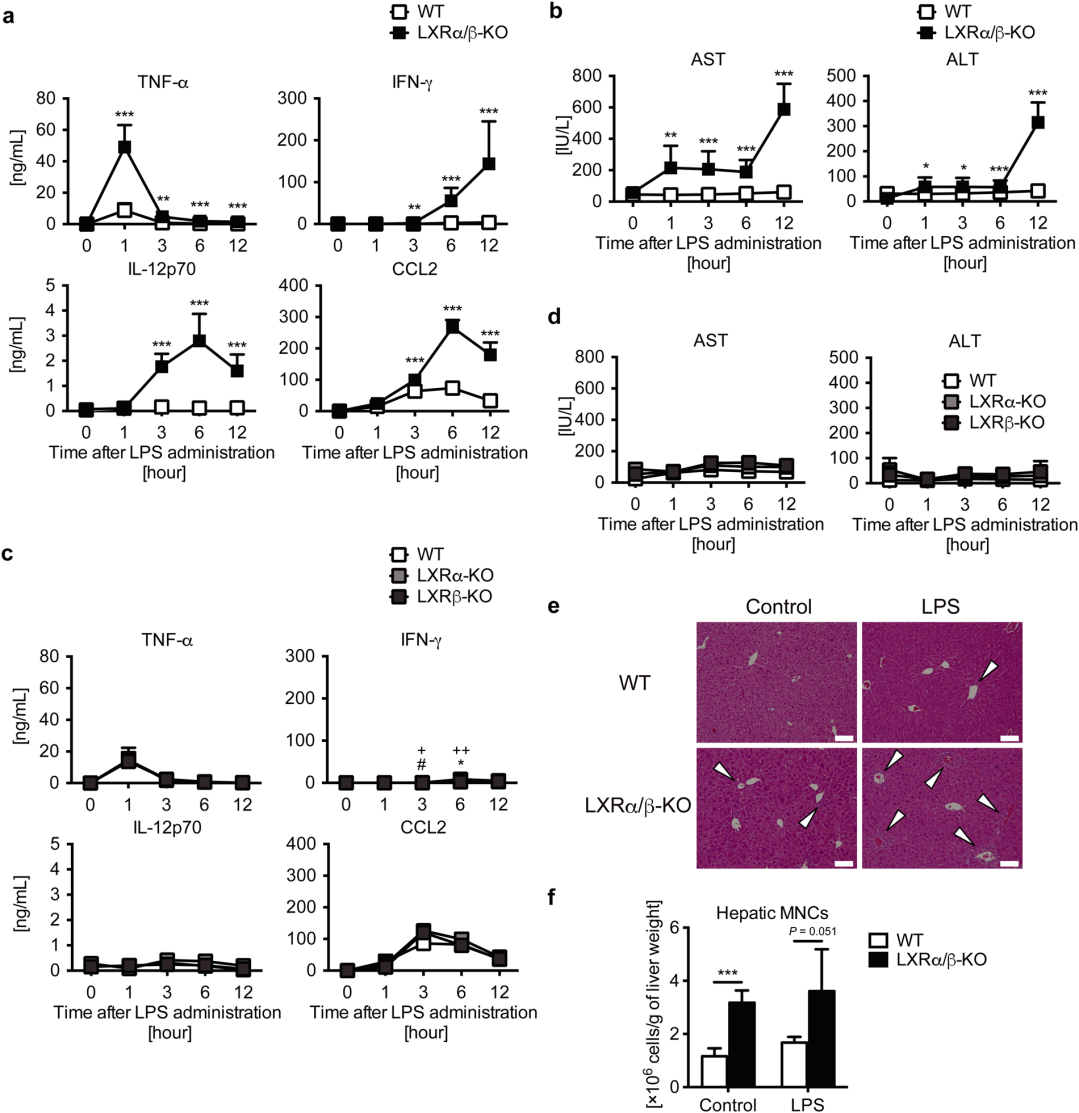
LXR deletion exacerbates LPS-induced acute hepatic inflammation in mice. LPS (2.5 mg/kg) was administrated to WT, LXRα-KO, LXRβ-KO and LXRα/β-KO mice via intravenous injection (**a**, **b**: WT, n = 9; LXRα/β-KO, n = 3; (**c**,**d**): WT, n = 4; LXRα-KO, n = 4; LXRβ-KO, n = 5; (**f**): WT, n = 4; LXRα/β-KO, n = 4). (**a**) Plasma TNF-α, IFN-γ, IL-12p70 and CCL2 levels were measured by ELISA. (**b**) Plasma aspartate aminotransferase (AST) and alanine aminotransferase (ALT) levels. Two-way ANOVA analysis shows that values between WT and LXRα/β-KO in (**a**) and (**b**) were statistically significant (two-way ANOVA). *P < 0.05; **P < 0.01; ***P < 0.001 compared to WT at the same time point (Student’s *t* test). (**c**) Plasma TNF-α, IFN-γ, IL-12p70 and CCL2 levels were measured by ELISA. (**d**) Plasma AST and ALT levels. *P < 0.05 (WT versus LXRα-KO); ^#^*P* < 0.05 (WT versus LXRβ-KO); + *P* < 0.05; ++ *P* < 0.01 (LXRα-KO versus LXRβ-KO) (Student’s *t* test). (**e**) Liver histology of WT or LXRα/β-KO mice after treatment of control (PBS) or LPS (2.5 mg/kg) for 12 hours. Scale bar = 100 μm. White arrows indicate infiltrated immune cells in periportal area. (**f**) The number of hepatic MNCs per g of liver weights in control (PBS) or LPS-treated WT or LXRα/β-KO mice (12 hours). ***P < 0.001 (Student’s *t* test).

Finally, to verify the ability of LXRs to regulate acute inflammation *in vivo*, we injected LPS intravenously into WT and LXRα/β-KO mice, and measured inflammation and liver injury markers in plasma. Consistent with mRNA expression in hepatic MNCs, LPS treatment strongly increased plasma levels of TNF-α, interferon γ (IFN-γ), IL-12p70, and CCL2 in LXRα/β-KO mice compared to WT mice (Fig. 8a). In LXRα/β-KO mice, TNF-α levels were elevated one hour after LPS administration, IL-12p70 and CCL2 levels peaked at 6 hours, and IFN-γ reached peak levels at or after 12 hours (Fig. 8a). Treatment with LPS (2.5 mg/kg) increased plasma levels of aspartate aminotransferase and alanine aminotransferase in LXRα/β-KO mice but not in WT mice (Fig. 8b). Next, we examined the effect of LPS on LXRα or LXRβ single KO mice. Consistent with findings on constitution of F4/80^+^CD11b^+^ Kupffer cells/macrophages (Fig. 3a), plasma levels of TNF-α, IL-12p70 and CCL2 in LXRα-KO and LXRβ-KO mice were comparable with WT mice, and IFN-γ levels were only slightly increased in LXRα-KO and LXRβ-KO mice compared to WT mice (Fig. 8c). LPS-induced liver injury was observed in LXRα-KO and LXRβ-KO mice (Fig. 8d), but much milder than LXRα/β-KO mice (Fig. 8b). Liver histology showed that LPS treatment slightly increased immune cell infiltration in the liver of WT mice and induced greater inflammation in the periportal area of the liver of LXRα/β-KO mice (Fig. 8e). The total number of hepatic MNCs tended to be elevated after LPS stimulation in LXRα/β-KO liver compared to WT liver, although it was not significant due to large variation (Fig. 8f). Therefore, both LXRα and LXRβ are necessary for protection from acute liver inflammation.

## Discussion

In this study, we investigated the role of LXRs in hepatic immunity in mice. First, we examined the expression of LXRα and LXRβ in mouse hepatic MNCs. While LXRα mRNA levels in hepatic MNCs were similar to those in the whole liver, LXRβ was expressed in hepatic MNCs more abundantly than in whole liver and at slightly higher levels than LXRα in hepatic MNCs (Fig. 1). F4/80^+^ Kupffer cells/macrophages were also expressed both LXRα and LXRβ (Fig. 1). Hepatic MNCs exhibited LXR target gene expression in a ligand-dependent manner both *in vitro* and *in vivo* as well as in isolated peritoneal macrophages (Fig. 1). The effect of LXR ligand on *Abca1* induction was weaker in hepatic MNCs than peritoneal macrophages. GW3965 tended to increase *Abca1* expression in hepatic MNCs but this difference was not statistically significant. Since GW3965 is less potent in LXRα activation than T0901317^28^ and GW3965 and T0901317 interact with LXRα and LXRβ differently^29,30^, higher concentrations of GW3965 may be required for significant induction of *Abca1* in hepatic MNCs. Further studies are needed to elucidate the underlying mechanism of cell type-selective and ligand-dependent LXR activation. LXRα is selectively expressed in Kupffer cells and spleen macrophages compared to other tissue-resident macrophages, and LXR target genes are highly expressed in these cells^17^. Thus, LXRα and LXRβ are expressed in hepatic immune cells, particularly monocytes/macrophages including Kupffer cells. Next, we compared the cell populations and function of hepatic MNCs in WT mice and LXR-deficient mice. The total number of hepatic MNCs was drastically elevated in LXRα/β-KO mice (Fig. 2), and F4/80^+^CD11b^+^ cells but not F4/80^+^CD11b^−^ or F4/80^+^CD68^+^ cells were increased in the liver of LXRα/β-KO mice (Fig. 3). These results indicate that bone marrow-derived Kupffer cells/macrophages are increased in the liver of LXRα/β-KO mice. A-Gonzales *et al*. also reported leukocyte infiltration^31^, but Schuster *et al*. did not observe infiltration of CD11b^+^ macrophages in the liver of LXRα/β-KO mice^22^. This discrepancy may be caused by differences in genetic background and/or rearing conditions. F4/80^+^CD11b^+^ hepatic MNCs are recruited to the liver from bone marrow in the setting of acute liver inflammation induced by injection of *Escherichia coli* or LPS^3^. LXR ablation in mice may increase sensitivity to intestinal bacterial translocation or LPS toxicity. LXRα/β-KO mice have increased hepatic cholesterol levels^22^, and cholesterol overload also increases F4/80^+^CD11b^+^ hepatic MNCs^10^. Increased cholesterol levels were observed not only in the liver but also in hepatic MNCs of LXRα/β-KO mice (Fig. 4). These findings suggest that innate immune signals, such as endotoxin exposure and cholesterol-induced cell damage, recruit CD11b^+^ macrophages in the liver of LXRα/β-KO mice.

Gene expression analysis showed that LXR-deficient hepatic MNCs have increased M1 macrophage markers and decreased M2 macrophage marker expression (Fig. 5). Inflammatory cytokine genes were also induced more effectively in these cells treated with LPS or CpG-DNA compared to WT MNCs (Fig. 7), consistent with recruitment of F4/80^+^CD11b^+^ MNCs with proinflammatory M1 markers in the liver. Interestingly, mRNA levels of *Cd68* were elevated in both F4/80^+^CD11b^+^ cells and F4/80^+^CD11b^−^ cells in the liver of LXRα/β-KO mice (Fig. 5). Although CD68 is used as a surface marker of resident Kupffer cells, it is also localized in the cytosol and expressed in bone marrow-derived CD11b^+^ monocytes/macrophages^3,32^. Gene expression of *Cd68* may be regulated differently from its membrane expression. LXR deletion elevated *Ccl2* mRNA expression in the whole liver and F4/80^+^CD11b^+^ Kupffer cells/macrophages (Fig. 5). However, there was no difference in *Ccl2* expression in WT and LXRα/β-KO MNCs. Hepatic MNCs isolated with our experimental method contain F4/80^+^ Kupffer cells/macrophages, natural killer cells, natural killer T cells, T lymphocytes and B lymphocytes. The whole liver samples, except for MNCs, include hepatocytes, stellate cells and vascular endothelial cells. CCL2 upregulated in hepatocytes and hepatic stellate cells mediates obesity-induced hepatic inflammation^33,34^. CCL2 is also increased in hepatocytes and Kupffer cells in mice with alcoholic liver injury^35^. Additionally, LPS treatment induces CCL2 expression in murine liver sinusoidal endothelial cells^36^. LXR activation decreases LPS-induced CCL2 production in mouse primary microglia and astrocytes^37^. In contrast, LXR activation induces TLR4 expression in human blood monocyte-derived macrophages but not in mouse bone marrow-derived macrophages and pretreatment with LXR agonist enhances secretion of CCL2 and TNF-α by LPS in human macrophages^16^. Thus, LXRs may regulate CCL2 expression through a species-specific and/or cell type-specific mechanism. Increased CCL2 in hepatocytes stimulates recruitment of myeloid cells expressing C-C chemokine receptor 2, a receptor for CCL2^34^. These findings suggest that increased CCL2 expression in hepatocytes and, to a lesser extent, F4/80^+^CD11b^+^ Kupffer cells/macrophages induces further recruitment of F4/80^+^CD11b^+^ bone marrow-derived Kupffer cells/macrophages with M1 markers in the liver of LXRα/β mice. LXRs may regulate cellular communication between hepatocytes and bone marrow-derived Kupffer cells/macrophages in hepatic immunity. Further analysis is required to characterize hepatic immune cells, including MNCs other than Kupffer cells/macrophages and immune cells other than MNCs, involved in LXR-mediated immune regulation in detail.

In cultured isolated hepatic MNCs, LXR activation suppressed LPS-induced proinflammatory cytokine expression (Fig. 6). Similar results have been reported in various immune cells, such as mouse peritoneal macrophages, intestinal CD11b^+^ immune cells, and rat cultured Kupffer cells^14,27,38^. On the other hand, expression of inflammatory cytokines was dramatically increased in MNCs isolated form the liver of LXRα/β-KO cells (Fig. 7). Thus, LXRs regulate both recruitment of cytokine-producing F4/80^+^CD11b^+^ cells and inflammatory cytokine production in immune cells. In contrast, Fontaine *et al*. reported that LXR activation enhances LPS responses in human macrophages^16^. LXR agonist treatment induces TLR4 expression in human blood monocyte-derived macrophages but not in mouse bone marrow-derived macrophages, and 48-hour pretreatment with LXR agonist enhances but 6-hour pretreatment decreases LPS-induced secretion of CCL2 and TNFα in human macrophages^16^. We treated hepatic MNCs with LXR agonists for 18 hours before LPS stimulation (Fig. 6). The discrepancy between our results and those of Fontaine *et al*. may be due to (1) a species difference between mouse and human in LXR induction of genes, such as *Tlr4*, (2) different cell types (hepatic MNCs versus blood monocyte-derived macrophages), or (3) different pretreatment with LXR agonist (18 hours versus 24 or 48 hours). Regulatory functions of LXRs in cytokine production and acute liver injury were revealed in experiments comparing WT, LXRα-KO, LXRβ-KO or LXRα/β-KO mice (Fig. 8). Among these mice, only LXRα/β-KO had an apparent acute liver injury phenotype, such as elevated levels of proinflammatory cytokines, aspartate aminotransferase and alanine aminotransferase in plasma (Fig. 8). These results are consistent with elevation of F4/80^+^CD11b^+^ cells only in LXRα/β-KO mice (Fig. 3). These findings indicate that both LXRα and LXRβ are involved in the regulation of acute hepatic immune responses.

This study has several limitations. First, we used conventional LXRα/β-KO mice. It remains unknown whether LXRα/β-KO F4/80^+^CD11b^+^ is autonomously activated or whether LXRα/β-KO hepatocytes induce activation of F4/80^+^CD11b^+^ cells. Analysis of mice with conditional deletion of LXRα, LXRβ or both in selective cells, such as hepatocytes and macrophages, is needed. Coculture experiments using cells from WT and LXRα/β-KO mice may also be useful to elucidate the interaction of these cells. Second, we observed increased F4/80^+^CD11b^+^ cells in the liver of LXRα/β-KO mice. Although these cells are suggested to be recruited from bone marrow, irradiation experiments and/or bone marrow transplantation can provide clues to the origin of hepatic F4/80^+^CD11b^+^ cells. Third, cholesterol levels were elevated in hepatic MNCs from LXRα/β-KO mice. We recently reported that high fat and high cholesterol diet feeding increases total hepatic MNCs and F4/80^+^CD68^+^CD11b^+^ cells in LXRα-KO mice^39^. Although these findings suggest that cholesterol accumulation induces hepatic immune activation, further investigation is needed to elucidate the detailed mechanisms. In conclusion, bone marrow-derived F4/80^+^CD11b^+^ Kupffer cells/macrophages with proinflammatory M1 markers are increased in the liver of LXRα/β-KO mice, and hepatic inflammation and injury are exacerbated in these mice. LXRs are involved in the regulation of hepatic immune reactions. Future studies are needed to elucidate the role of LXRs as oxysterol receptors and lipid metabolism regulators in hepatic immunity.

## Methods

### Mice

C57BL/6J mice were obtained from Nihon CLEA (Tokyo, Japan). *Nr1h3*^−/−^ (*Lxr*α^−/−^, LXRα-KO), *Nr1h2*^−/−^ (*Lxr*β^−/−^, LXRβ-KO) and *Nr1h3*^−/−^;*Nr1h2*^−/−^ (*Lxr*α^−/−^;*Lxr*β^−/−^, LXRα/β-KO) mice were kindly provided by Dr. David J. Mangelsdorf (University of Texas Southwestern Medical Center at Dallas, TX)^40,41^, and were backcrossed with C57BL/6 J mice for at least ten generations. Mice were maintained under controlled temperature (23 ± 1 °C) and humidity (45–65%) with a 12-h light, 12-h dark cycles, and with free access to water and chow (CE-2; Nihon CLEA). Experimental samples were collected from male mice between 8 and 16 weeks of age. The experimental protocol adhered to the Nihon University Rules concerning Animal Care and Use and was approved by Nihon University Animal Care and Use Committee.

### Reagents

T0901317 [N-(2,2,2-trifluoro-ethyl)-N-[4-(2,2,2-trifluoro-1-hydroxy-1-trifluoromethyl- ethyl)-phenyl]-benzenesulfonamide]] was purchased from Cayman Chemical Company (Ann Arbor, MI), 24(*S*),25-epoxycholesterol was from Enzo Life Science (Farmingdale, NY), *Escherichia coli*-derived LPS was from Sigma-Aldrich (St. Louis, MO), and CpG-DNA was from Hycult Biotech (Plymouth Meeting, PA). GW3965 (3-[3-[*N*-(2-chloro-3-trifluoromethylbenzyl)-(2,2-di- phenylethyl)amino]propyloxy]phenylacetic acid hydrochloride) was kindly provided from Dr. Hiroyuki Miyachi (the University of Tokyo, Japan).

### Isolation of mouse peritoneal macrophages and hepatic MNCs

For isolation of peritoneal macrophages, mice were injected intraperitoneally with 1 mL/20 g of body weight of 3% thioglycolate (Thermo Fisher Scientific, Waltham, MA). After 4 days, macrophages were elicited with phosphate-buffered saline and cultured in RPMI 1640 medium containing 10% FBS as previously described^42^. Hepatic MNCs, including Kupffer cells, were isolated with collagenase digestion and Percoll gradient centrifugation as previously described^11^. Isolated peritoneal macrophage and hepatic MNCs were plated in 24-well plate (1 × 10^6^ cells/well) for LXR ligand treatment.

### Reverse transcription and quantitative real-time polymerase chain reaction

Total RNAs from tissue or cell samples were prepared with the acid guanidine thiocyanate-phenol/chloroform method as described previously^43^. cDNAs were synthesized using the ImProm-II reverse transcription system (Promega Corporation, Madison, WI). Quantitative real time polymerase chain reaction was employed with the ABI PRISM 7000 sequence detection system (Thermo Fisher Scientific) or the StepOnePlus real-time PCR system (Thermo Fisher Scientific) using Power SYBR Green PCR Master Mix (Thermo Fisher Scientific). Primer sequences were as follows: *Adgre1* (encoding F4/80), 5′- GCA TCA TGG CAT ACC TGT TC-3′ and 5′-GAG CTA AGG TCA GTC TTC CT-3′; *Itgam* (encoding CD11b), 5′-TCC TGT ACC ACT CAT TGT GG-3′ and 5′-GGG CAG CTT CAT TCA TCA TG-3′; *Cd68*, 5′-CTG CTG TGG AAA TGC AAG CA-3′ and 5′-TGG TCA CGG TTG CAA GAG AA-3′; *Arg1* (encoding arginase-1), 5′-GTT CTG GGA GGC CTA TCT TA-3′ and 5′-CCA AGA GTT GGG TTC ACT TC-3′; *Retnla* (encoding resistin-like molecule α), 5′-CGA AGA CTC TCT CTT GCA CT-3′ and 5′-TCC CAA GAT CCA CAG GCA AA-3′; *Cd163*, 5′-CTC TTG GTT TGT GGA GCC AT-3′ and 5′-AAG ACC TCT CCT CTT GAG GA-3′; *Ccl2*, 5′-ATG CAG GTC CCT GTC ATG CTT-3′ and 5′-AGC TCT CCA GCC TAC TCA TTG-3′. Other primer sequences were as previously reported^44–46^. The mRNA values were normalized with the mRNA levels of *Ppib* (encoding cyclophillin B) or *Gapdh* (encoding glyceraldehyde-3-phosphate dehydrogenase).

### Liver histology

Liver sections were fixed with 10% neutral buffered formalin (Muto Pure Chemicals, Tokyo, Japan) for 24 hours and embedded with paraffin. The embedded tissues were cut into 5 μm width, de-paraffinized, stained with hematoxylin and eosin (Sakura Finetek Japan, Tokyo, Japan), washed with ethanol and xylene, and mounted with the Histofine Mousestain kit (Nichirei Corporation, Tokyo, Japan). Immunohistochemistry was performed with anti-F4/80 antibody (Thermo Fisher Scientific), the Histofine Simple Stain Mouse MAX-PO (Rat) kit (Nichirei Corporation), and the ImmPACT DAB substrate solution (Vector laboratories, Burlingame, CA). For double immunostaining, frozen sections of liver samples were cut into 5 μm width, fixed with acetone, incubated with anti-CD11b antibody (Thermo Fisher Scientific) overnight and with goat anti-rat IgG (H + L)-AP (BioFX Laboratories, Owings Mills, MD) for 1 hour, and stained with the New Fuchsin Substrate kit (Nichirei Coorporation) for red staining. Next, sections were incubated with fluorescein isothiocyanate-conjugated (FITC-conjugated) anti-F4/80 antibody (Thermo Fisher Scientific) overnight and with anti-FITC antibody conjugated with HRP (Antibodies-online.com, Aachen, Germany) for 1 hour, stained with 3,3′-diaminobenzidine tetrahydrochloride (Dojindo Laboratories, Kumamoto, Japan) for brown staining or the Abcye HistoGreen Sustrate kit for Peroxidase (Eurobio, Les Ulis, France) for green staining, and counterstained with hematoxylin.

### FACS analysis

For mRNA expression analysis, hepatic MNCs were incubated with anti-CD16/32 Fc blocker (Thermo Fisher Scientific) for 15 min at 4 °C, stained with FITC-conjugated anti-F4/80 (Thermo Fisher Scientific) and phycoerythrin-Cy5-conjugated (PE-Cy5-conjugated) anti-CD11b, and sorted into F4/80^+^CD11b^+^ cells and F4/80^+^CD11b^−^ cells using a cell sorter (SH800S; Sony Corporation, Tokyo, Japan). For flow cytometric analysis, hepatic MNCs were incubated with anti-CD16/32 Fc blocker (Thermo Fisher Scientific) for 15 min at 4 °C, stained with FITC-conjugate anti-F4/80 (Thermo Fisher Scientific), biotin-conjugated anti-CD68 (Bio-Rad Laboratories, Hercules, CA), PE-Cy5-conjugated anti-CD11b and PE-streptavidin (Thermo Fisher Scientific), and analyzed using a flow cytometer (Cytomics FC500; Beckman Coulter, Indianapolis, IN). For intracellular cytokine analysis, hepatic MNCs were stimulated with LPS (1 μg/mL) or CpG-DNA (10 μg/mL) in the presence of BD GolgiPlug (1 μL/mL of medium; BD Biosciences, San Jose, CA) for 4 hours. Cells were harvested, incubated with anti-CD16/32 Fc blocker, stained with the LIVE/DEAD Fixable Near-IR Dead Cell Stain Kit (Thermo Fisher Scientific), FITC-anti-F4/80, biotin-anti-CD68, Pacific Blue-anti-CD11b (BioLegend, San Diego, CA), PE-Cy5-streptavidin, PE-anti-TNF-α (Thermo Fisher Scientific) and allophycocyanin-conjugated anti-IL-12 (BD Biosciences), and analyzed with a flow cytometer (BD FACSVerse; BD Biosciences). Cell frequencies were analyzed with FlowJo 10.4 (Tree Star, Ashland, OR).

### Measurement of lipid contents

Whole liver tissues or isolated hepatic MNCs were homogenized in 0.3 M phosphate buffer (pH 6.0) on ice, and lipids were extracted as previously reported^47^. Cholesterol and triglyceride levels were measured with Cholesterol E-Testwako (Wako Pure Chemical Industries, Osaka, Japan) and Triglyceride E-Testwako (Wako Pure Chemical Industries), respectively.

### LPS-induced acute hepatic inflammation

LPS (2.5 mg/kg) was injected intravenously into mice. Plasma was collected at 0, 1, 3, 6 and 12 hours after LPS injection, and used for measurement of concentrations of aspartate aminotransferase, alanine aminotransferase (Wako Pure Chemical Industries), and cytokines. Cytokine concentrations were determined with ELISA kits (R&D Systems, Minneapolis, MN).

### Statistical analysis

Data are presented as means ± S.D. We performed one-way ANOVA followed by Tukey’s multiple comparisons to analyze data of two or more groups, unpaired two-group Student’s *t* test to analyze data of two groups (WT versus LXR-α/β-KO), or two-way ANOVA to analyze the influence of two different factors using Prism 6 (GraphPad Software, La Jolla, CA).

### Data availability

All relevant data are available from the corresponding author upon request.

## Electronic supplementary material

Supplementary information
