# Supplementary material for: Liver X receptors regulate hepatic F4/80+CD11b+ Kupffer cells/macrophages and innate immune responses in mice
Source: Sci Rep. 2018 Jun 18;8:9281. doi: 10.1038/s41598-018-27615-7 (PMC6006359; doi:10.1038/s41598-018-27615-7)
Supplement: Supplementary file 1 — Supplementary information [file 41598_2018_27615_MOESM1_ESM.pdf]

**Supplementary Information**

# Liver X receptors regulate hepatic F4/80$^{+}$CD11b$^{+}$ Kupffer cells/macrophages and innate immune responses in mice

Kaori Endo-Umeda[1], Hiroyuki Nakashima[2], Shihoko Komine-Aizawa[3], Naoki Umeda[1], Shuhji Seki[2], and Makoto Makishima[1,*]

[1]Division of Biochemistry, Department of Biomedical Sciences, Nihon University School of Medicine, 30-1 Oyaguchi-kamicho, Itabashi-ku, Tokyo 173-8610, Japan
[2]Department of Immunology and Microbiology, National Defense Medical College, 3-2 Namiki, Tokorozawa, Saitama 359-8513, Japan
[3]Division of Microbiology, Department of Pathology and Microbiology, Nihon University School of Medicine, 30-1 Oyaguchi-kamicho, Itabashi-ku, Tokyo 173-8610, Japan
*Corresponding author. e-mail: makishima.makoto@nihon-u.ac.jp

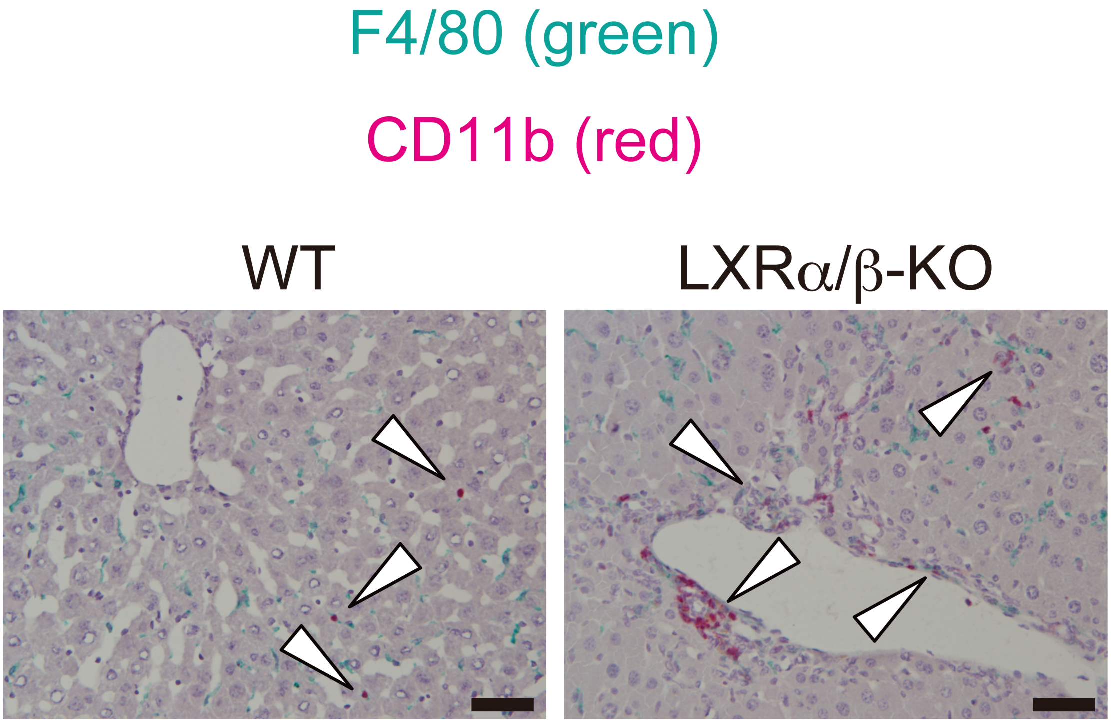


Supplementary Figure 1. Double immunostaining of F4/80 (green) and CD11b (red) in the liver of WT and LXRα/β-KO mice. The open triangles indicate F4/80$^+$CD11b$^+$ cells. Scale bar = 50 μm.
